# Knowledge and potential impact of the WHO Global code of practice on the international recruitment of health personnel: Does it matter for source and destination country stakeholders?

**DOI:** 10.1186/s12960-016-0128-5

**Published:** 2016-06-30

**Authors:** Ivy Lynn Bourgeault, Ronald Labonté, Corinne Packer, Vivien Runnels, Gail Tomblin Murphy

**Affiliations:** Telfer School of Management, University of Ottawa, 1 Stewart St, Ottawa, ON K1N6N5 Canada; School of Epidemiology, Public Health and Preventive Medicine, University of Ottawa, Ottawa, ON Canada; WHO/PAHO Collaborating Centre Health Workforce Planning and Research, School of Nursing, Dalhousie University, Halifax, NS Canada

**Keywords:** Destination countries, Migration of health workers, Source countries, WHO Code

## Abstract

The WHO Global Code of Practice on the International Recruitment of Health Personnel was implemented in May 2010. The present commentary offers some insights into what is known about the Code five years on, as well as its potential impact, drawing from interviews with health care and policy stakeholders from a number of ‘source’ and ‘destination’ countries.

## Background

The World Health Organization (WHO) Global Code of Practice on the International Recruitment of Health Personnel was implemented by the World Health Assembly in May 2010. The present study offers some insights into key stakeholders’ perspectives and the potential impact of the Code five years since its implementation. This international study considers the perspectives of a range of health care and policy stakeholders from a number of ‘source’ countries, including the Philippines, India, South Africa, and Jamaica. These insights are supplemented by those from similar stakeholders in a number of ‘destination’ countries, including Canada, the USA, the United Kingdom, and Australia, as part of earlier and related studies [1–3].

## The impact of previous codes

Despite good intentions, the WHO and earlier similar codes have so far failed to regulate active or passive recruitment or to stem the tide of migrating health workers [4, 5]. Part of the problem is related to scope and coverage. The United Kingdom Code of Practice for the International Recruitment of Healthcare Professionals [6], for example, did not initially cover private sector employers, but because the National Health Service recruits from private employers, its own Code was circumvented. Another problem results from the lack of an enforcement mechanism. These limitations hold true for the WHO Code, despite it covering the widest possible range of employers in both the public and private sectors and including non-governmental organizations, professional associations, and regional health authorities.

## Knowledge of the WHO Code – ‘destination’ countries

Awareness of international agreements on best practice should incentivize such practice. Thus, it is essential that key stakeholders in important source and destination countries are aware of the Code. Previous research in Canada and other destination countries, which involved interviews with 189 stakeholders, suggests a general lack of knowledge of any Code, WHO or otherwise, amongst those directly responsible for health worker recruitment, namely local employers and regional health authorities [1, 7, 8]. Indeed, presentations to some of these Canadian stakeholders about the knock-on effects of their efforts to recruit local health care workers in such exporting source countries as the Philippines and India were often met with shocked responses. Justifications for their active recruitment of, for example, nurses in the Philippines and doctors in India, were based on claims of local ‘shortages’, which is more accurately described as maldistribution, in the destination country (Canada), and ‘surpluses’, which more accurately reflects under- and unemployment, in the two source countries. There was little or no awareness that, in many source countries, health worker to population ratios (i.e., physicians, nurses, and midwives) fall below the WHO’s critical threshold guidelines of 2.28 per 1000. Although the four source countries we studied have varying health worker population ratios (none of which currently fall under the critical threshold, although India is very close), they still remain significantly lower than Canada’s ratios, which are well above the guidelines [9].

## Knowledge of the WHO Code – ‘source’ countries

A more recent study of the migration of health workers from the Philippines, South Africa, Jamaica, and India, which involved interviews with 144 stakeholders across health, education, labour, and migration sectors, included questions regarding their knowledge of and the impact of the WHO Code on their human resources for health challenges [3]. Additionally, scoping reviews of the published and grey literature and policy documents dating from the year 2000 to date were conducted to analyse what had been written locally about the WHO and previous Codes. In two cases – Jamaica and the Philippines – within-country policy dialogues were held with over two dozen stakeholders in each case.

Although the Code is intended to assist in protecting the integrity of a state’s health systems with respect to health workers, as well as migrating health workers themselves, and to provide recruitment guidance to public and private sector employers, knowledge of the WHO Code was minimal among those interviewed and all but absent in policy documents. Participants had little to offer with respect to the Code, apart from complaining that “it has no teeth.” From the Indian case study, one informant stated, “The codes have no importance in practice. [The] Government of India wants people to migrate because they get foreign exchange [remittances].” This reveals how departments within some source country governments can have conflicting interests. Another commented, “The Government of India does not object to foreign agencies and governments recruiting Indians for foreign jobs.” Therefore, a stronger domestic concern about health worker shortages and/or migration, and specifically a better knowledge of the Code, could help reduce both health worker recruitment and migration. A key finding from the study is that any direct effects of the Code to lever policies that respond to increasing trends in health worker migration are minimal or are difficult to assess.

## Making the code work – insights from key international organization representatives

To augment these local case studies with data from a broader international perspective, representatives from key international agencies with mandates to address international health worker migration were consulted. These interviews revealed a keen awareness of the Code, but raised issues regarding its lack of impact on health worker migration trends and potential policy responses, similar to those voiced by local stakeholders. These international stakeholders further stressed a need to improve shared responsibility and international cooperation on migration policy and development, including bilateral and trade agreements. Further, they questioned the lack of clarity in the Code regarding which organisation or government department holds responsibility for its implementation, monitoring, and reporting at the national level; this reflects a general concern that the WHO needs to encourage and promote better collaboration and shared responsibility for the Code between different ministries (e.g., health, justice, finance, employment) in both source and destination countries. Similar to previous findings [4, 7, 10], as one respondent noted, “…a lot of the action [on the Code] needs to happen at the country level. People just don’t [act on it], it’s not a priority.”

## Conclusion

While the Code has raised some awareness of problems associated with migration and staff shortages in source countries, knowledge and implementation of the Code is variable across levels of governance in both the source and destination countries in the studies discussed. Simply put: the Code does not have prominence in those countries that need it most, namely those still lacking sufficient health workers and experiencing ongoing out-migration of those they train. The ‘push’ of inadequately financed or administered systems in many source countries remains deeply problematic; yet, this also remains largely unaddressed in the Code. Until the conversation on both the ‘push’ and ‘pull’ across countries exporting and importing health workers deepens, the Code risks having little impact on its laudable goal of ensuring ethical and equitable health worker migration.
